# Developing non-response weights to account for attrition-related bias in a longitudinal pregnancy cohort.

**DOI:** 10.23889/ijpds.v7i3.1925

**Published:** 2022-08-25

**Authors:** Tona Pitt, Kamala Adhikarit, Shainur Premji, Sheila McDonald

**Affiliations:** 1 University of Calgary; 2 Alberta Health Services; 3 University of York

## Objectives

The prospective cohort study design is ideal for examining diseases of public health importance. A main source of potential bias for longitudinal studies is attrition. In this study, we compare the performance of two models developed to predict sources of attrition and develop weights to adjust for potential bias.

## Approach

This study used the All Our Families longitudinal pregnancy cohort of 3351 maternal-infant pairs. Logistic regression models were developed to predict study continuation versus drop-out from baseline to the three-year data collection wave.

Two methods of variable selection took place. One method used previous knowledge and content expertise while the second used Least Absolute Shrinkage and Selection Operator (LASSO). Model performance for both methods were compared using area under the receiver operator curve values (AUROC) and calibration plots. Stabilized inverse probability weights were generated using predicted probabilities. Weight performance was assessed using standardized differences with and without weights (unadjusted estimates).

## Results

LASSO and investigator prediction models had good and fair discrimination with AUROC of 0.73 (95% Confidence Interval [CI]: 0.71 – 0.75) and 0.69 ( 95% CI: 0.67 – 0.71), respectively. Calibration plots and non significant Hosmer-Lemeshow Goodness of Fit Tests indicated that both the LASSO model (p = 0.10) and investigator model (p = 0.50) were well-calibrated.

Unweighted results indicated large (>10%) standardized differences in 15 demographic data variables (range: 11% - 29%), when comparing those who continued in study with those that did not. Weights derived from the LASSO and investigator models reduced standardized differences relative to unadjusted estimates, with ranges of 0.1% - 5.3% and 0.3% - 12.7%, respectively.

## Conclusion

The data-driven approach produced robust weights that addressed non-response bias more than the knowledge-driven approach. The data driven approach, did, however still require content knowledge in how data were grouped, combined, or split. The weights can be applied to analyses across multiple waves of data collection to reduce bias.

